# Cardio-Diagnostic Assisting Computer System

**DOI:** 10.3390/diagnostics10050322

**Published:** 2020-05-19

**Authors:** Galya Georgieva-Tsaneva, Evgeniya Gospodinova, Mitko Gospodinov, Krasimir Cheshmedzhiev

**Affiliations:** Institute of Robotics, Bulgarian Academy of Science, 1113 Sofia, Bulgaria; jenigospodinova@abv.bg (E.G.); mitgo@abv.bg (M.G.); cheshmedzhiev@gmail.com (K.C.)

**Keywords:** computer system, heart rate variability, cardiovascular diseases, Holter records, arrhythmia, heart failure, syncope, mathematical analysis

## Abstract

The mathematical analysis and the assessment of heart rate variability (HRV) based on computer systems can assist the diagnostic process with determining the cardiac status of patients. The new cardio-diagnostic assisting computer system created uses the classic Time-Domain, Frequency-Domain, and Time-Frequency analysis indices, as well as the nonlinear methods (Poincaré plot, Recurrence plot, Hurst R/S method, Detrended Fluctuation Analysis (DFA), Multi-Fractal DFA, Approximate Entropy and Sample Entropy). To test the feasibility of the software developed, 24-hour Holter recordings of four groups of people were analysed: healthy subjects and patients with arrhythmia, heart failure and syncope. Time-Domain (SDNN < 50 ms, SDANN < 100 ms, RMSSD < 17 ms) and Frequency-Domain (the spectrum of HRV in the LF < 550 ms^2^, and HF < 540 ms^2^) parameter values decreased in the cardiovascular disease groups compared to the control group as a result of lower HRV due to decreased parasympathetic and increased sympathetic activity. The results of the nonlinear analysis showed low values of (SD1 < 56 ms, SD2 < 110 ms) at Poincaré plot (Alpha < 90 ms) at DFA in patients with diseases. Significantly reducing these parameters are markers of cardiac dysfunction. The examined groups of patients showed an increase in the parameters (DET% > 95, REC% > 38, ENTR > 3.2) at the Recurrence plot. This is evidence of a pathological change in the regulation of heart rhythm. The system created can be useful in making the diagnosis by the cardiologist and in bringing greater accuracy and objectivity to the treatment.

## 1. Introduction

Diseases of the heart and blood vessels are one of the major problems of medicine today. The mathematical analysis of cardiac examination data can be used in diagnostics to clarify the diagnosis, to predict future diseases and to carry out effective treatment. One of the methods of diagnosing cardiovascular disorders about the tasks of preventive medicine is the analysis of HRV information, which is based on a mathematical analysis of the dynamics of changes in heart rate [1,2]. HRV is currently one of the popular methods in non-invasive cardiology, sports medicine, and physiology. The term "heart rate variability" refers to natural fluctuations in the number of intervals between successive heartbeats. The method is based on the recognition and measurement of time intervals between the R peaks (RR intervals) of electrocardiogram (ECG) signals (or P peaks of photoplethysmogram (PPG) signals—PP intervals) and the subsequent analysis of the obtained numerical values-of the studied parameters or graphical images, using various mathematical methods [3]. Heart rate reflects not only the state of the cardiovascular system but also the whole organism, as it is a major biomarker for the functioning of the autonomic nervous system and reflects the balance between the two compartments—the sympathetic and the parasympathetic. Increased activity of the sympathetic nervous system leads to a decrease in HRV, and the inverse activity of the parasympathetic division of the nervous system leads to an increase in HRV [4].

In recent years, the proportion of medical software products has grown rapidly because healthcare is an important part of many people’s lives and their use has the potential to improve the quality and effectiveness of medical services for the ongoing monitoring of patients’ health. The benefits of using software products in medicine are: providing remote medical consultation (which is particularly useful for people living in small, non-hospital towns), functional, database-based diagnostics processed automatically without the participation of a doctor, and to notify the user of any deviations in the values of the investigated parameters [5]. Because of such software products, diagnosis becomes more accurate and faster, and the prescribed treatment is more effective.

Research that is currently of interest to health professionals is largely related to the use of methods for mathematical analysis of HRV, as one of the most accessible and fairly informative ways to analyse and evaluate the general condition of the human body. The combination of simple, non-invasive information-gathering technology obtained from ECG and PPG devices [6] with full automation of the calculations and the ability to physiologically interpret the results obtained through the application of software products is the basis for the widespread clinical use of HRV technology in the diagnostic process, medical screening and treatment.

### 1.1. Background

HRV is widely used as a biomarker for the diagnosis and prognosis of several cardiovascular diseases. Reynders et al. [7] investigate changes in HRV (of ECG data) parameters in Multiple Sclerosis Disease. The studies done include the parameters in the time domain.

Kim et al. [8] include HRV obtained via an ECG (five minutes, Lead II channel) in the creation of a predictive model useful in the diagnosis of cardiovascular disease. The methods used are: determining parameters in Time and Frequency Domain, Poincaré plot indicators, Approximate Entropy, Hurst exponent, and exponent α of the 1/f spectrum.

Park et al. [9] examine the impact of six activities (sitting, standing, walking, ascending, resting, and running) using HRV (ECG data obtained in a controlled laboratory environment) to evaluate the impact of human activity on a person’s health.

Investigation of the differences between several cardiac diseases using mathematical methods is researched in [10]. The following methods are used: Approximate Entropy, Bi-Spectral Entropies, Recurrence Entropy, and Sample Entropy. Based on the methods studied, the authors present a Composed Integrated Index to help distinguish between normal and abnormal classes more accurately.

Several authors have been working to accurately identify atypical heart contractions. Inan et al. [11] use a wavelet-based method to detect premature ventricular contractions that are characteristic of some types of arrhythmias. The method is applied to public databases.

In their study, the authors of [12] point out the benefits of using computer systems for analysis and diagnosis in medicine: reducing time and improving accuracy in diagnosis. A review of scientific publications concerning computer-aided cardiac data analysis has been made and the differences between an offline and an online system are outlined.

HRV is also suitable for analysis in patients with other conditions (such as diabetes) [13]. Computer tools for processing diagnostic information are increasingly being used and preferred in the study of biomedical information obtained from patients with cardiovascular disease [14]. In clinical cardiology, computerized diagnostic procedures are being used today to aid diagnosis and treatment. Early diagnosis is crucial for a good prognosis of any disease [15], this also applies to the early diagnosis of heart disease, which has a high prevalence rate.

Software systems for the analysis of the HRV, developed in recent years, mainly offer the classical analysis in the time domain and the frequency domain. Of the nonlinear methods, the Poincaré plot is predominantly proposed, other software systems only offer DFA, and a small number of systems offer both analyses. Some of these software systems offer a graphical user interface, allowing them to be used by a wider range of users. Most systems are desktop applications and are created in MATLAB (Kubios [16], CODESNA_HRV [17], KARDIA [18], SinusCor [19], POLYAN [20] etc.), and gHRV [21] was created in the Python programming language, rHRV [22] was developed in the "R" programming language. These software systems use the following methods:

- Kubios: Time and Frequency analysis, Poincaré plot, DFA; ApEn, Recurrence plot, Entropy;
- CODESNA_HRV: Time and Frequency analysis, Entropy;
- KARDIA: Time and Frequency analysis, DFA;
- SinusCor: Time and Frequency analysis, Time-Frequency analysis (Fast Fourier Transform and AutoRegressive method);
- POLYAN: Time and Frequency analysis, Nonlinear methods;
- gHRV: Time and Frequency analysis, Poincaré plot, Entropy, Fractal Dimension;
- rHRV: Time and Frequency analysis, Poincaré plot.

Many mathematical methods for analysing and evaluating HRV can be successfully used to assist diagnosis in determining the cardiac status of patients. Graphic representation of the results of the application of such methods is a synthesized assessment of the current state of patients for the studied period. Application of mathematical methods through modern high-performance portable computer systems enables rapid, inexpensive assessment of cardiac activity in all conditions. They are at the basis of modern cardiology telemedicine and the ability to consult specialists in real time, regardless of the patient’s location.

The review of existing software for analysis and evaluation of HRV shows that there is currently no uniform format for the presentation of input data (RR interval series). In many cases, available software products do not allow the use of data obtained from different ECG and PPG devices.

The creating of a computer system to support the diagnostic process, integrating all proven methods of HRV analysis time domain (statistical and geometrical methods), frequency domain, and nonlinear means (Poincaré plot, DFA, Multi-Fractal DFA, Hurst R/S, entropy) is a task that would help improve the efficiency of using mathematically based tools for cardiac data analysis.

### 1.2. The Purpose of This Article

The purpose of this article is to present a computerized system for assisting cardio diagnosis, consisting of developed algorithms, software for mathematical analysis of HRV and a database consisting of patients’ Holter records, and numerical and graphical results for different disease groups to support the diagnosis. The development is based on established mathematical methods for the processing and analysis of diagnostic cardiac information for patients to evaluate the condition of the cardiovascular system and determine the current or predict future cardiac disease.

For validation of the developed cardio-diagnostic assisting system, named by the authors HeartAnalyzer, four groups of subjects were examined: healthy and patients with different cardiovascular diseases (arrhythmia, heart failure, and syncope) by applying linear and nonlinear mathematical methods. The system allows autonomous computer processing and analysis of patient data to monitor their cardiac status in real time. In case of deviation in the results, they can seek professional advice, help, and treatment from a cardiologist.

## 2. Materials and Methods

### 2.1. A Computer System for Analysis and Evaluation of HRV

The motivation for creating a new computer system is determined by the need to increase the efficiency in the development of automated systems to support the activity of diagnosing the functionality of the human body, which is achieved through the development of a set of algorithms and software for analysis and evaluation of HRV. The HeartAnalyzer offers, in addition to the ability to analyze and evaluate HRV and study the dynamics of changes in cardiovascular activity, creating an archive for each patient that allows the treating physician to view the patient’s data and monitor his or her condition. In addition to the results of the patient’s analysis, the database will also store graphic information specific to the various diseases to assist the physician in diagnosing the disease. The presented software system is in the process of testing. It is a computerized system that is created solely for the purpose of research and has no commercial purpose.

**Figure 1:**
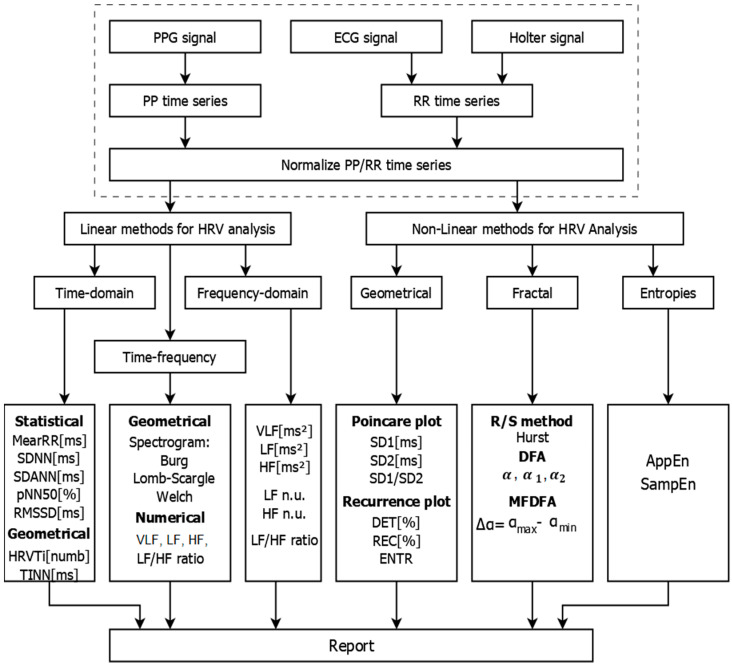
Block diagram of the cardio-diagnostic HeartAnalyzer.

Figure 1 shows the block diagram of the created diagnostic HeartAnalyzer, which operates in the following three main modes:

- Cardiology registration via ECG, PPG and Holter devices and receipt of PP/RR time series;
- Mathematical analysis of the recorded data by applying linear and nonlinear methods. The results of the analysis are presented in tabular and graphical form;
- Creating a Report based on the results obtained, which can be stored in the patient database for later review and/or printing. In addition to the patient data, the database contains graphical information obtained through the graphical methods of analysis of HRV characteristics of various cardiovascular diseases.

The software checks that the RR/PP time series are normalized and, if not, normalizes them. The normalization is the following: it removes any RR/PP intervals that are shorter than 330 ms or longer than 1.2 s, and those that are 25% shorter or 25% longer than the median of the previous five RR/PP intervals.

Mathematical analysis is performed on the normalized RR/PP time series by applying the following linear and nonlinear methods:

- Linear methods: Time-Domain, Frequency-Domain, and Time-Frequency analysis;
- Nonlinear methods: Poincaré plot, Recurrence plot, Hurst R/S method, DFA, Multi-Fractal DFA, AppEn and SampEn.

**Figure 2:**
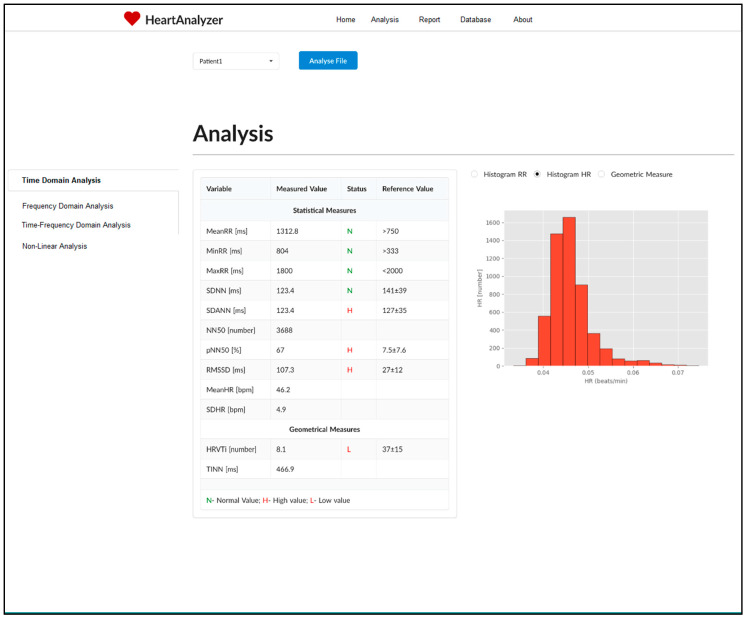
The home page of the proposed diagnostic HeartAnalyzer.

Figure 2 shows the home page of the HeartAnalyzer diagnostic system. It allows you to select an input file and then begins analysing the data. The results of the analysis and evaluation are displayed by selecting the appropriate button (Time-Domain, Frequency-Domain, Time-Frequency and Nonlinear analysis), by default the results of Time-Domain analysis are displayed. The results are presented in two ways: tabular and graphical.

In addition to the values of the investigated parameters, in the table shows and the reference values from the Time-Domain and Frequency-Domain analysis. These methods are standardized by the European Society of Cardiology and the North American Society of Pacing and Electrophysiology [23], with the limits of norm-pathology known. When the value of the parameter under study is within the limits of the reference values, in the Status field indicates N, and when the value is below the lower limit—L and above the upper limit—H. The graphical results are displayed in the right of the table by selecting the appropriate radio button.

### 2.2. Linear Methods for HRV Analysis

#### 2.2.1. Time-Domain Analysis

HRV time-domain analysis is based on the statistical analysis of changes in the duration of consecutive normal NN (RR) intervals obtained from ECG signals. This type of analysis is performed for long records (24 hours) through statistical and graphical measurements. The statistical analysis calculates the following parameters [24]:

- SDNN (ms)—this parameter calculates the standard deviation from the average duration of RR intervals over the entire study period. It is used to evaluate total HRV and especially its parasympathetic component. The longer the study lasts, the more total HRV accumulates, so it is necessary that the compared signals have the same duration;
- SDANN (ms)—it defines the standard deviation from the average length of RR intervals by calculating the 5-minute segments. The registration period is split when a 24-hour ECG recording is used. This parameter is used to evaluate the low frequency components of HRV;
- SDNN index—determines the average of standard deviations from the average duration of RR intervals for all 5-minute periods divided by the observation period;
- RMSSD (ms)—determines the root mean square difference between the duration of adjacent RR intervals. This parameter reflects the fast, high frequency variability changes;
- NN50—the number of the pairs of consecutive NN intervals differing by more than 50 ms obtained over the entire recording period;
- pNN50—the percentage of consecutive intervals that differ by more than 50 ms. Because this parameter is determined by adjacent intervals, it reflects fast, high frequency variability changes.

The Time-Domain analysis parameters are integral to the sample and describe the average statistical characteristics of the digital performance of the entire signal or fragments thereof. The values of the statistical parameters depend on the duration of the data surveyed and on what hours of the day and under what conditions the ECG records were made.

Time-Domain heart rate analysis not only allows you to determine the values of HRV statistics but can also present them graphically.

Histograms construction refers to geometric methods. They allow graphical representation of the distribution of RR interval series. Geometric methods are less affected by the quality of the recorded data and can be considered as an alternative to statistical measurements. However, the recorded ECG signals should be at least 20 minutes in length, therefore short-term records cannot be estimated using the geometric methods. The geometric methods calculate the following parameters:

- TINN—the distribution of RR intervals is approximated to a triangle and its base is measured in milliseconds. The essence of the algorithm is the following: the histogram is conventionally represented as a triangle, the base of the triangle is calculated by the formula: b = 2A/h, where h is the largest number of RR intervals, and A is the area of the whole histogram, i.e., the total number of all RR intervals analysed. This parameter avoids taking into account the RR intervals associated with artifacts and extrasystoles that form additional peaks and domes of the histogram;
- HRV triangular index—this parameter plot a histogram of RR intervals at 7.8125 ms (1/128 s). The total number of RR intervals is divided by the peak height of the histogram. This index reflects total HRV and is directly proportional to parasympathetic activity.

Using the histogram, the relationship between the total number of identified RR intervals and their variation is calculated. For the HRV triangular index, the highest peak of the histogram is taken as the triangle point, the basis of which corresponds to the quantitative value of the variability of the RR intervals. The height of the triangle corresponds to the most frequently observed duration of the RR intervals, and its face corresponds to the total number of all RR intervals involved in its construction. The HRV triangular index parameter provides an estimate of the overall HRV.

#### 2.2.2. Frequency-Domain Analysis

Spectral analysis of cardiac data (showing the mode of action of the heart and the entire cardiovascular system) presents the distribution of frequencies present in the NN interval series as a mathematical sum of regular sinusoids of different amplitudes.

The evaluation of HRV in frequency analysis is performed by determining the power spectral density (PSD) characteristic, which is investigated in different frequency ranges. Cardiac recordings lasting from 5 to 30 minutes are obtained under steady-state conditions using an electrocardiograph and are defined as short-term data records. Long-term Holter monitoring of cardiac activity records in real non-stationary conditions (having a duration of 24 to 72 hours and even up to 2 weeks) is defined as long-term data records. There are some differences in the way long-term and long-term data records are analysed, which were defined in the Common European American Standard in 1996 (recommendations have been made for studies to use certain frequency bands) [23].

Following physiological reasons, the spectral parameters in the frequency range are determined and distributed in the following frequency ranges [23]:

- Very low frequency—VLF: from 0.003 Hz to 0.04 Hz;
- Low frequency—LF: from 0.04 Hz to 0.15 Hz;
- High frequency—HF: from 0.15 Hz to 0.4 Hz.

This article uses the Welch mathematical method to determine the spectral parameters, which is a modification of the traditional periodogram and is proven to be an effective spectral analysis method. The investigated cardiac series that have undergone preprocessing and consisting of normal intervals are divided into overlapping segments to reduce the high dispersion of the periodogram.

With the Welch method, the data located at the end of the time series studied are processed to obtain a smaller weighting factor than the data located in the center. Assuming that each segment has M elements, then a modified periodogram for one of the overlapping segments is calculated by the formula [25]:

(1) $$P_{Mod,X}\left(f\right)=\frac{1}{M.U}{\left|\sum_{n=0}^{M-1}x_{i}(n)w\left(n\right)e^{-j2\pi fn}\;\right|}^{2},$$

where:

n=0 to M−1,

i=0 to L−1,

*M* – length of the blocks;

$x_{i}\left(n\right)=x\left(n+iD\right)\;$—*i-th* block;

*f*—frequency;

*w*—window function;

*iD*—offset of i-th block;

U is the normalizing factor [25]: $U=\frac{1}{M}.\sum_{n=0}^{M-1}w^{2}\left(n\right)$.

The modified Welch periodogram is applied sequentially to all segments, after which the average PSD of the studied segments is determined [25]:

(2) $$P_{Welch}\left(f\right)=\frac{1}{L}\sum_{i=0}^{L-1}P_{Mod,\;\;i}\left(f\right),$$

*L*—Number of segments.

#### 2.2.3. Time-Frequency Analysis

Time-Frequency Analysis, Wavelet-Based Method

Using the time-frequency method of analysis, graphical representations of ordinary and logarithmic spectrograms of spectral density can be constructed using three popular and efficient methods: the Burg method, the LombScargle method, and the Wavelet based method. Spectrograms are color graphs that show the frequency distribution (vertical axis) versus time (horizontal).

The spectrograms give a visual idea of signal strength by using a pre-selected color palette. The dark blue color is an indication of the absence of a frequency in the frequency spectrum. Light blue to yellow to red indicates an increase in the power of the corresponding frequency in the energy spectrum. The highest frequency power is indicated by a dark red. Dense horizontal lines mark the boundaries of the frequency ranges.

Methods for constructing spectrograms:

- Burg method—this method uses an autoregressive model of a different order, spline interpolation, Heming window, and window overlap apply;
- LombScargle method—the method calculates a non-normalized Lomb–Scargle periodogram;
- Wavelet method—based on the application of wavelet theory methods; applies wavelet interpolation of the investigated data, uses different wavelet bases (Morlet, Dobeshi, bi-orthogonal wavelets, and other wavelet bases) and calculates a continuous wavelet spectrum.

The results in the frequency domain are assumed to be calculated according to the variability standard for a selected five-minute segment of the input data. Cubic spline wavelet interpolation is used in the present work. 

The numerical results give information about the values of the spectrum of data in absolute units—$ms^{2}$, in percentages and normal units for the three frequency bands VLF, LF, and HF. Frequency peaks for each frequency range are determined. The sympathetic balance index LF/HF is also calculated. Investigations are conducted based on the removed trend data during preprocessing.

The results of the wavelet spectral analysis and the determined frequency characteristics in this work are calculated for real cardiac recordings.

### 2.3. Nonlinear Methods for HRV Analysis

Traditional techniques for HRV analysis in time and frequency domains are often not sufficient to characterize the complex dynamics of cardiac rhythm since the mechanisms involved in cardiovascular regulation are likely to interact in a nonlinear manner [26]. It has been shown that the heart of a healthy person can act very randomly, and the reduction of HRV and the appearance of a pronounced periodicity can be associated with several diseases [27]. Guided by this concept, the use of nonlinear mathematical methods in the analysis of HRV may provide additional, useful information to evaluate the functional state of the organism, but also monitor its dynamics and the occurrence of pathological conditions with a sharp decrease in HRV and high likelihood of sudden death [28]. According to the recommendations of the European Society of Cardiology and the North American Society of Pacing and Electrophysiology [23], the study of the applicability of nonlinear HRV methods is currently one of the most important research areas.

#### 2.3.1. Geometric Nonlinear Methods

The correlation rhythmogram (spectrogram) obtained by the Poincaré plot allows a compact representation of the entire series of cardio intervals, no matter how long the study lasted—several minutes or many hours [28]. In the rectangular coordinate system, each pair of RR intervals (previous and next) has coordinates (x, y), where x is the value of the RR_n_ interval and y is the value of RR_n + 1_. The formation of the graph yields a segment of points whose center is located on the line of identity. The identity line is a graph of the function x = y (RR_n_ = RR_n+1_). On the identity line of the correlation, rhythmograms are those cardio intervals whose duration is approximately equal to the duration of the previous interval. If the point corresponding to a cardio interval is located above the line of identity, this indicates how much longer the heart interval is than the previous one, i.e., x < y (RR_n_ < RR_n+1_). Accordingly, if the point is below the identity line, this indicates that the RR_n+1_ interval is shorter than the RRn interval (RR_n_ > RR_n+1_). Therefore, the shape of the point segment (RR_n_; RR_n+1_) on the graph reflects the change in the duration of the RR intervals, i.e., the variance. If an ellipse with a longitudinal and a transverse axis is placed on the graph constructed by Poincaré plot, the following parameters can be determined [29]:

- Ellipse length (SD2 [ms] parameter)—corresponds to long-term variability of RR intervals and reflects total HRV;
- Ellipse Width (SD1 [ms] parameter)—represents the scattering of the dots perpendicular to the identity line and is associated with rapid variations between heart beats;
- The SD1/SD2 ratio reflects the relationship between short- and long-term HRV.

The main features that are used for visual analysis of HRV using the Poincaré method are: the shape, size of the main segment of points, and the symmetry of points concerning the identity line.

The *shape of the segment of points* is categorized for the different functional states of the person [28,29]:

- The healthy subject’s graph has one major segment of points, which has the shape of a comet with a narrow bottom and gradually expanding to the top;
- The chart of the sick subject has the form of a torpedo, a fan or a complex form (consisting of several segments) depending on the type of disease.

Graphics constructed using Poincaré plot can be quantified by placing an ellipse on the graphical form. The *size of the segment of points* is characterized by the parameters: length and width of the ellipse.

The length of the ellipse reflects the involvement of the non-respiratory components in the formation of the common HRV and is determined by the parameter SD2 [29].

The width of the ellipse takes into account the long-term variations and demonstrates the contribution of respiratory arrhythmias to the total HRV and is determined by the parameter SD1 [29]. 

*The symmetry of the segment of points* defined concerning the line of identity is the next factor to be considered in the visual analysis of the resulting graph. Symmetry shows the equilibrium state of the HRV and the absence of rhythmic disturbance, and asymmetry is the opposite—for the presence of such.

The size of the segment of points and the symmetry of the points in the graph are categorized for the different functional states of the person [29]:

- The graphic of a healthy subject has a clear ellipse;
- If the graph looks like a compressed segment of dots, then the narrow "compressed" ellipse means low HRV and is an indicator of a disease state;
- If the length and width of the ellipse are approximately equal and it approaches a circle, in this case, the HRV is low, which is an indicator of the disease state;
- If the points in the graph are symmetrical relative to the identity line, then there is no rhythm disturbance;
- If the points in the graph are asymmetric relative to the identity line, then the patient has rhythmic disturbances.

Another promising method for studying the physiological fluctuations of non-stationary processes is the Recurrence plot, which allows visualizing certain regularities in the models of the complex non-stationary fluctuations [30,31,32]. The basis of the method is the construction and analysis of a recurrence diagram. The algorithm for construction and analysis by the Recurrence plot consists of the reconstruction of the phase space. There are two major factors that play an important role in the reconstruction of the phase space of a dynamic system: embedding dimension m and time delay τ. The parameter m is determined by the False Nearest Neighbours (FNN) method [33,34] and τ is determined by the Mutual information function [35]. Based on these two parameters, the phase space is constructed and Euclidean distances between vectors (system states) are analysed. If the distances between the points i and j are below the threshold value ε, i.e., $\varepsilon =\mathrm{SDNN}\sqrt{m}$, where SDNN is the standard deviation of the normal RR intervals, then a point with the coordinates i and j is placed in the recurrence diagram. In this way, a pattern of points forming vertical and diagonal lines appears on the recurrence diagram. The diagonal lines reflect the reappearance of a sequence of system states and are manifestations of the coincidence of system behaviour in two different time sequences. Vertical lines arise because of the stability of a condition over some time. The analysis of the topology of the recurrence diagram allows us to classify the following processes:

- Homogeneous processes with independent random values;
- Processes with slowly changing parameters;
- Periodic or oscillating processes, etc.

The numerical analysis of the recurrence diagrams consists of determining the following parameters:

- Recurrence rate (REC%)—this parameter reflects the level of recurrence, indicating the probability of finding a recurring point in the RR series, that is, determining the probability of a recurrence of the condition. This variable ranges from 0% to 100%.
- Determinism (DET%)—this parameter is a characteristic of the predictability of the system. It is defined as the ratio between the number of recurrent points located on diagonal lines and the total number of recurrent points.
- Lmax, Lmean indicators reflect the maximum and average length of the diagonal lines, assuming that Lmin = 2. The Lmax parameter is associated with the largest Lyapunov exponent (LLE) [36,37].
- ENTR—this parameter is related to Shannon entropy.

The advantage of the Recurrence plot is that it allows the properties of the studied processes to be represented as a geometric figure. This method is a tool for detecting hidden dependencies in the observed RR interval series.

#### 2.3.2. Fractal Methods

It is common knowledge that RR intervals are nonlinear and non-stationary time series, with much of the information encoded in the dynamics of their fluctuations. These fluctuations have an internal structure with fractal (self-similar) properties that can be observed at different time intervals. Therefore, fluctuations can be measured by fractal and multifractal indicators. One of the main properties of fractal processes is: self-similarity. The degree of self-similarity can only be determined by one parameter known as the Hurst parameter (H) [38,39]. The value of this parameter is in the range between 0 and 1. When 0 < H < 0.5, the process is antipersistence, which means that the upward trend in the past means a decline in the future and vice versa. If H > 0.5, the process is persistence and it has stable behaviour, i.e., the upward trend in the past remains in the future. The higher the value of this parameter, the stronger the trend. In the case of H = 0.5, no process trend is observed and it has Brown motion behaviour. Therefore, based on the value of the Hurst exponent, it is possible to predict and predict the future behaviour of the investigated RR signal. The following methods are used in this software to determine the Hurst parameter: Hurst R/S method, Detrended Fluctuation Analysis and Multi-Fractal Detrended Fluctuation Analysis (MFDFA).

Hurst R/S method is a process that requires processing a large amount of data. In this method, the RR interval series is divided into non-overlapping blocks, and for each block, the range R (n) and standard deviation S (n) are determined, where n is the number of points [40,41]. A linear regression model is constructed between the dependent variable Log_10_(R(n)/S(n)) and the independent variable Log_10_(block size). The method of least squares determines the regression coefficients:

- β_0_—the point at which the regression line intersects the ordinate;
- β_1_—the slope of the regression line.

The value of the Hurst parameter is equal to the slope of the regression line: H = β_1_. If the value of H is in the range (0.5, 1.0), then the process under study is fractal.

Detrended Fluctuation Analysis is suitable for studying both stationary and non-stationary processes in terms of statistical self-similarity [42,43]. The DFA determines the signal fluctuation coefficient that is related to the Hurst exponent. To determine the "profile" of the study signal with a length of N, it is divided into no overlapping blocks of length s. The local trend for each segment s is calculated, using the least squares method and determination of the sums for the segments. For different values of the parameter, *s* is calculated the fluctuation function: $F\left(s\right)\sim s^{\alpha }.$ The graphical dependence of Log_10_(Fs) vs Log_10_(s) is plotted and the value of the fluctuation index α is determined from the slope of the line. The DFA exponent α and the Hurst parameter H are related by:

- H = α if 0< α <1;
- H = α-1 if α ≥ 1.

If the value of parameter α is less than one, then its value is equal to the Hurst exponent. The value of the α parameter in the case of fractal signals is greater than 0.5 and less than 1. The greater the value of the H parameter, the more regular the process with a high degree of self-similarity.

The DFA method shows typically two ranges of scale invariance, which are quantified by two separate scaling exponents, α_1_ and α_2_, reflecting the short-term and long-term correlation. The short-term fluctuation is characterized by the slope α_1_ obtained from the (log_10_(s), log_10_(F_s_)) graph within range [4,16] and long-range fluctuation is characterized by the slope α_2_ obtained from the range [17].

Multi-Fractal Detrended Fluctuation Analysis is a method to examine the self-similarity of a nonlinear, chaotic, and noisy time series [44,45,46,47]. Fractal processes are of two main classes: monofractal and multifractal. The monofractal process is homogeneous in the sense that it has the same scale properties locally as well as globally. These processes can be described with only one value of the Hurst exponent or with one value of the fractal dimension. In contrast to these signals, multifractal signals can be decomposed into a large number of homogeneous, fractal subsets that are characterized by a spectrum of local Hurst exponents. The multifractal approach us allows to describe a broad class of structurally more complex signals than those that are completely characterized by a single fractal dimension. This approach makes it possible to obtain new estimates that give an idea of the intrinsic, nonlinear, dynamic processes in the studied signals. MFDFA quantifies the presence or absence of fractal, correlation properties in the non-stationary signals studied.

#### 2.3.3. Entropies Methods

Approximate Entropy (AppEn) and Sample Entropy (SampEN) as nonlinear methods for analysing HRV. These methods determine the degree of irregularity of the RR time series. The low entropy values are characteristic of regular time series, while higher values are inherent in stochastic data [45].

### 2.4. Data Collection

The cardiology data used to conduct the study in this article were recorded using a Holter monitoring device at the Medical University of Varna, Bulgaria. The Holter device has been on the patients for about 10 hours in the morning under the supervision of a cardiologist, after which the patients were free to perform their daily routine. Registration of data under the conditions in which the individual usually lives is of particular importance, given a realistic picture of the conditions in which his or her heart problems occur. The Holter data are transferred to the server the next day after the Holter device has been removed. This study was approved by the Research Ethics Committee at Medical University—Varna, Bulgaria., Protocol/Decision No. 82, 28 March 2019. All participants were informed in advance of the research that would be done to them. The participants are from Varna, Bulgaria and are aged 49 to 68 years, men and women. Holter records have been made between April 2019 and March 2020. In addition, 229 Holter records were investigated, 14 of which were excluded due to concomitant diseases, and 3 records were excluded due to the assumption that the records were not correct (records contain corrupted data). The records are approximately 24 hours in length and have been reviewed for the correctness of data.

The 212 records studied were divided into groups according to their major cardiac disease, as determined by a cardiologist: Group 1—healthy controls (48 number volunteers who were not diagnosed with cardiac disease), Group 2—patients with arrhythmia (56 number), Group 3—patients with heart failure (59 number), and Group 4—patients with syncope (49 number).

### 2.5. Statistical Analysis

Continuous variables are reported as mean and standard deviation (SD). Results were expressed as mean ± SD unless indicated otherwise. *Statistical significance*. In the field of research, from the statistical point of view, it is important to choose the cut-off point below which, if the *p*-value falls, the parameter under study is considered statistically significant. In our study, if the *p*-value tested was less than or equal to 0.05 (5%), the result was considered statistically significant. All statistical analyses (performed between each heart disease group and the healthy control group) were performed using the *t*-test.

## 3. Results

### 3.1. Linear Methods for HRV Analysis

Table 1 shows the values of the studied parameters in Time-Domain analysis for the four studied groups. The statistical parameters SDNN, SDANN, and SDNN index reflect the analysis of consecutive RR intervals. The values of these parameters decreased in the cardiovascular disease groups compared to the control group. This approach eliminates random fluctuations in RR intervals, often associated with artefacts or the occurrence of arrhythmias and another cardiovascular disease. The standard [23] provides for the use of graphical methods for the evaluation of histograms and the calculation of parameter values: TINN and HRV triangular index. The value of the HRV triangular index parameter decreases in groups with cardiovascular disease compared with healthy controls.

**Figure 3:**
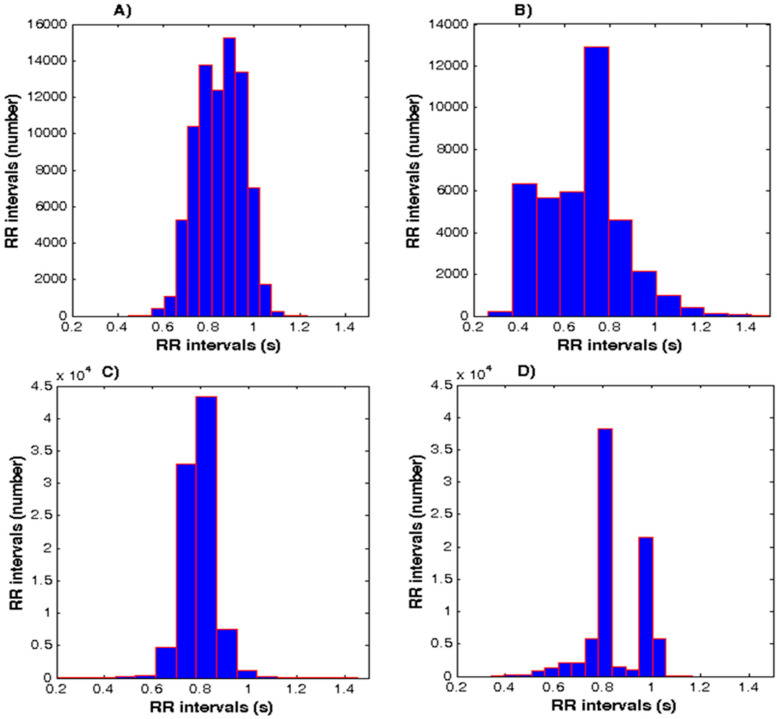
Histograms for healthy subject and patients with arrhythmia, heart failure, and syncope. (**A**) Healthy subject. (**B**) Patient with arrhythmia. (**C**) Patient with heart failure. (**D**) Patient with syncope.

Figure 3A–D shows the histograms of a healthy subject, a patient with arrhythmia, heart failure, and syncope. The graphs clearly show that there is a difference in the shape of the histograms in patients with cardiovascular disease and healthy subjects. The healthy controls are characterized by the central arrangement of the pillars in the diagram of the RR intervals with the localization of the highest pillars (fashion) in the range 0.7–1.0 s. Normal cardiac activity is characterized by asymmetrical, dome-shaped and dense histogram, the shape is similar in appearance to the Gaussian curve (Figure 3A). The asymmetric shape of the histogram shows the arrhythmic nature of the ECG. An example of such a histogram is shown in Figure 3B. In Figure 3C shows a histogram of a patient with heart failure. This histogram has a very narrow base and a pointed tip. Figure 3D shows the histogram of a patient with syncope. This chart is multifaceted and there are two pronounced peaks.

The considered parameters for Time-Domain analysis are highly correlated with each other, which is why the standard [23] proposes to use clinically the following five parameters: SDNN, SDANN, pNN50%, RMSSD and HRV triangular index, for which reference values are entered, i.e., the boundaries of norm-pathology are known.

**Figure 4:**
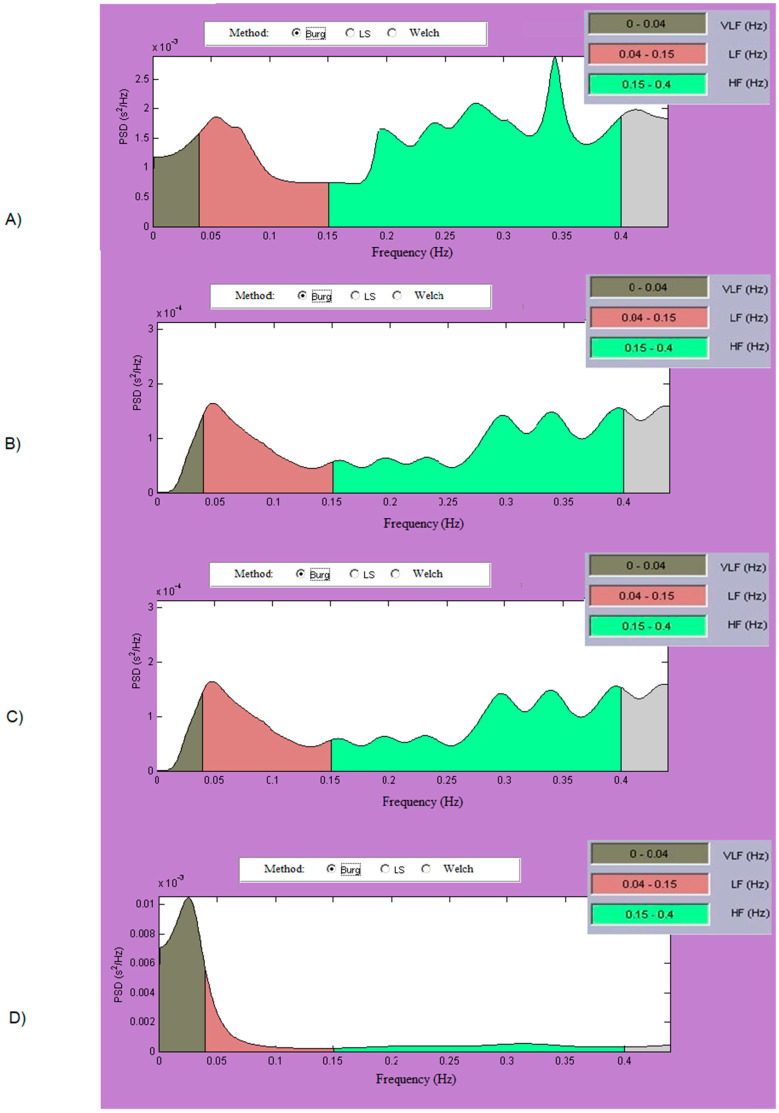
Frequency domain, PSD. (**A**) Healthy subject. (**B**) Patient with arrhythmia. (**C**) Patient with heart failure. (**D**) Patient with syncope.

The results of the studies performed in the Frequency domain on three patient groups and the healthy control group are summarized in Table 2. In the healthy control group (Figure 4A), high values of the spectral parameters can be established in the three tested frequency bands, with the LF Power/HF Power ratio reflecting the mean value for the studied group 1.52, which is within the range of values indicating good health status according to the variability standard.

Patients with arrhythmia (Figure 4B) have low LF and HF spectral components properties and the lowest LF/HF Power ratio (0.81 versus 1.52 for health group, *p*-value = 0.0001). These values indicate that arrhythmia as a disease significantly impairs the spectral characteristics of the heart rate signal, indicating that this disease impairs the body’s restorative powers and is an indicator of the severity of this common heart disease.

A significant decrease in VLF Power values was observed in patients with heart failure (Figure 4C) (11939.57 versus 13226.42 ms^2^ for health group, *p*-value = 0.0001). Reduced VLF Power in combination with other variables may indicate patients with heart failure who are at increased risk of future cardiovascular events. The values of the spectral components in the LF and HF area show a 2 to 3-fold decrease in heart failure patients compared to the healthy control group. This is most characteristic of conducted studies of patients with heart failure: slightly lowered VLF Power values, but several times lowered LF Power values (411.82 versus 1198.88 ms^2^ for health group) and HF Power (301.93 versus 791.03 ms^2^ for health group) areas. Studies on daily parts of the Holter record show even greater reductions in LF and HF Power values, up to five times lower than healthy (in some patients even greater). It can be concluded that the overall activity of the sympathetic nervous system is increasing.

Cardiac patients who have developed syncope (Figure 4D) have a marked increase in spectrum in VLF (17846.86 versus 13226.42 ms^2^ for health group). Syncope is a sudden loss of consciousness caused by a sharp decrease in heart rate and/or blood pressure. Assessment by gender, age, obesity, diabetes, and other physiological parameters showed no significant differences. This is most characteristic of patients undergoing syncope: have high VLF Power values (significantly higher than healthy) at the expense of rather low values in LF Power (486.26 versus 1198.88 ms^2^ for health group) and HF Power (534.35 versus 791.03 ms^2^ for health group) areas. VLF Power values reflect the activity of the sympathetic nervous system, parasympathetic rhythms, mechanisms of thermoregulation and the renin-angiotensin-aldosterone system. The LF/HF = 0.91 ratio indicates a disease according to the variability standard. A possible option is: high VLF Power reflects the sympathetic balance at rest in subjects who will receive syncope and so that prediction can be made of the occurrence of this event (based on high VLF Power above normal value). In normal units, the spectral components in LF and HF areas are determined (relative to the total power in these two areas).

Relative to the healthy control group, all LFnu and HFnu have segmental differences except for heart failure values. In heart failure, LFnu and HFnu (*p*-value > 0.05) cannot be considered as defining indicators for this group relative to healthy controls.

**Figure 5:**
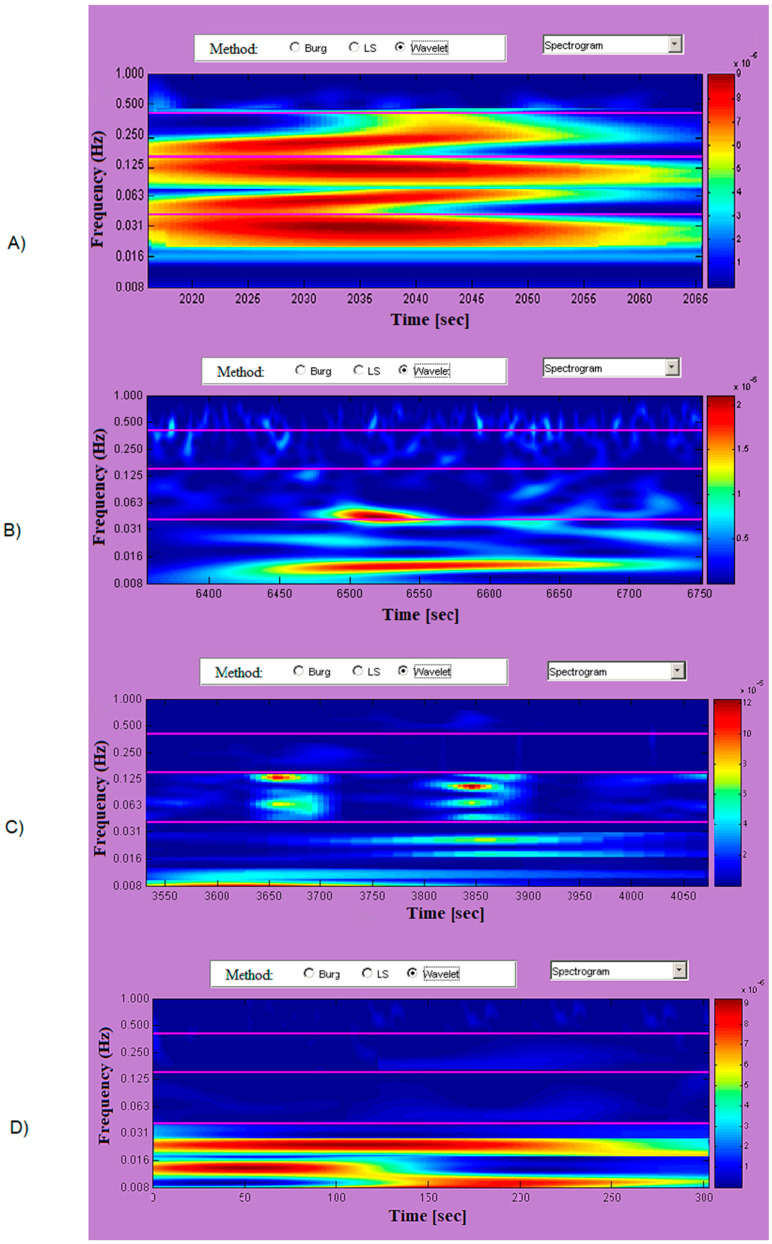
Time-Frequency spectrograms. (**A**) Healthy subject. (**B**) Patient with arrhythmia. (**C**) Patient with heart failure. (**D**) Patient with syncope.

The results in the time-frequency domain are presented as spectrograms obtained by applying a wavelet-based method. In healthy subjects, the power of the investigated signal is high in the three frequency bands depicted in Figure 5A through vast areas of red and orange fields. The spectrogram of Figure 5B is characteristic of patients in the study group diagnosed with arrhythmia. Moments of high variability (mainly in the LF region) are observed, followed by longer intervals of low variability (fields in blue).

The results of Figure 5C present a spectrogram of a patient with heart failure. The study group of patients showed high signal power values only in the VLF range. The other two frequency bands are characterized by low signal power values, which is indicative of HRV in HF and LH areas.

The power of the investigated signal is very low in all frequency ranges, in patients diagnosed with syncope (Figure 5D) across vast areas of blue. The spectrogram shows graphically low heart rate variability across all frequency ranges.

### 3.2. Nonlinear Methods for HRV Analysis

**Figure 6:**
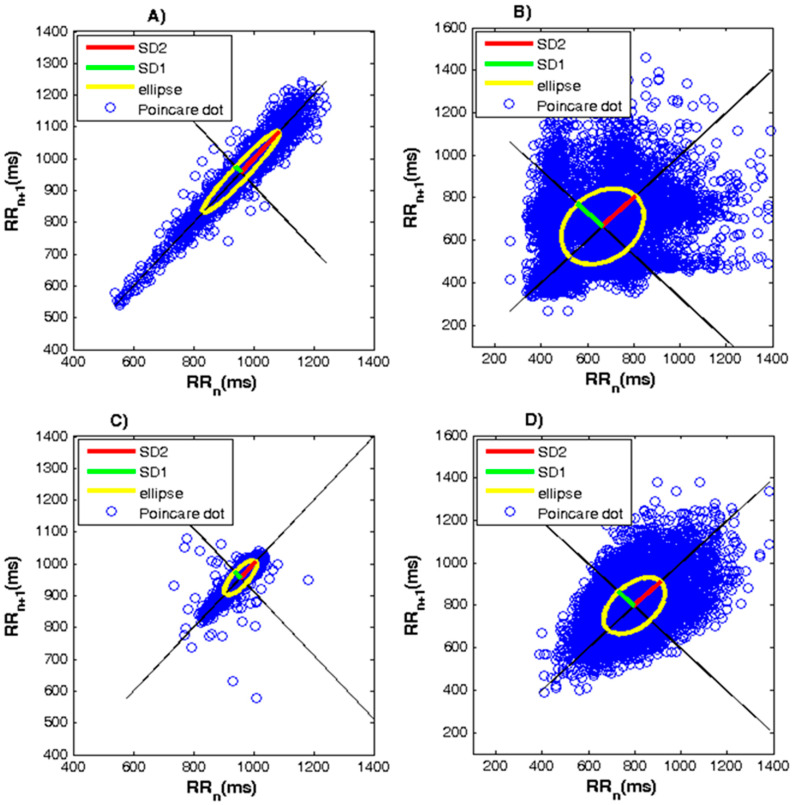
Poincaré plot for healthy subject and patients with arrhythmia, heart failure, and syncope. (**A**) Healthy subject. (**B**) Patient with arrhythmia. (**C**) Patient with heart failure. (**D**) Patient with syncope.

Poincaré plot. The quantitative characteristics of the SD1, SD2 parameters and the relationship between them for the study groups are shown in Table 3. The value of the SD1 parameter decreased in the three groups of patients compared to the healthy controls. This decrease was statistically significant (*p* < 0.0001). The value of SD2 decreased almost twice in patients with diseases compared to healthy controls (*p* < 0.0001). Based on the examined 24-hour Holter ECG records, Figure 6 shows the graphs constructed using the Poincaré method for a healthy subject and patients with arrhythmia, heart failure, and syncope. The healthy subject’s graph (Figure 6A) is shaped like a comet with a pointed bottom and gradually expanding to the top. The arrhythmia patient chart (Figure 6B) is in the form of a ventilator and the cardiac failure chart is strongly compressed (Figure 6B), while the syncope patient chart is a torpedo (Figure 6C). An ellipse is constructed for each diagram. In a healthy subject, the ellipse is clearly pronounced, whereas in patients with arrhythmia and syncope, the length and width of the ellipse are approximately equal and the ellipse approaches a circle. For the four graphs, the locations of the points in the segments relative to the identity line are symmetrical.

**Figure 7:**
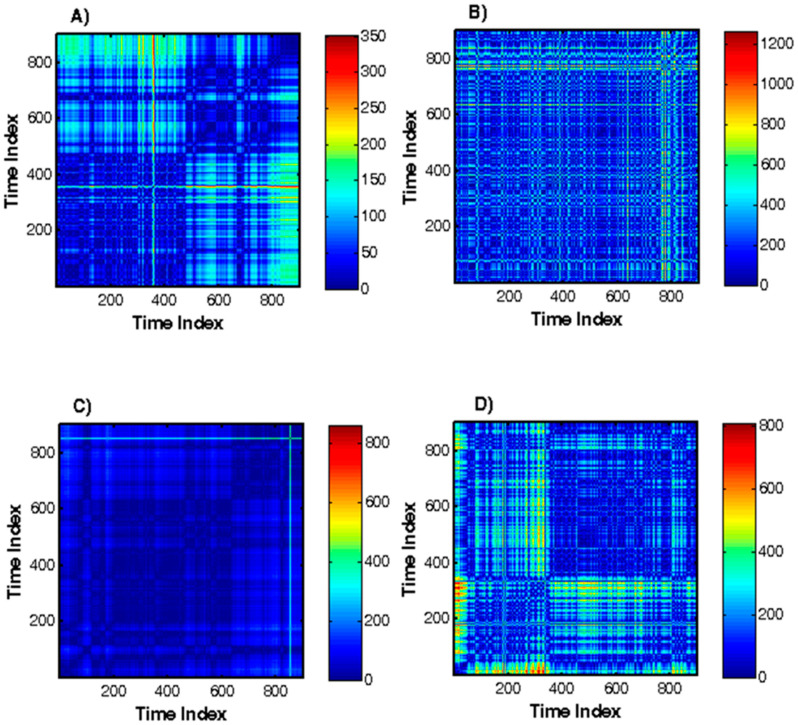
Recurrence plot for healthy subject and patients with arrhythmia, heart failure, and syncope. (**A**) Healthy subject. (**B**) Patient with arrhythmia. (**C**) Patient with heart failure. (**D**) Patient with syncope.

Recurrence plot. The results of the Recurrence Quantification Analysis (DET%, REC%, Lmax, ShanEn) are reported in Table 3. The values of the studied parameters are increased in the patients with arrhythmia, heart failure and syncope compared with healthy subjects with their statistical significance being *p* < 0.0001. Figure 7 shows the graphs obtained using the Recurrence plot for the first 10 minutes of the 24-hour Holter records for the four groups studied. For a healthy subject, the graph has fewer squares compared to patients with cardiovascular disease. This is evidence of a higher HRV. Anomalies such as arrhythmia and syncope have more squares in the graph showing the frequency of the signal being tested. In this case, HRV decreases. Similar results have been reported in [32]. The visual evaluation of the repetitive diagrams provides quick information on the behavior of the studied process. Reducing the complexity of the process (heart rhythm) and switching to periodicity is indicative of a pathological change in the regulation of heart rhythm. The cardiovascular disease significantly affects the dynamics of RR intervals, with HRV decreasing. The advantage of this method is that it can clearly illustrate the properties of the studied processes.

Hurst R/S method. Table 3 shows the values obtained for the Hurst exponent of the study groups. Hurst exponent is a marker for predictability of time series. The value of this parameter in the four groups studied has a high degree of self-similarity, with a statistical significance greater than 0.05. Therefore, this method cannot distinguish healthy subjects from patients with cardiovascular disease.

**Figure 8:**
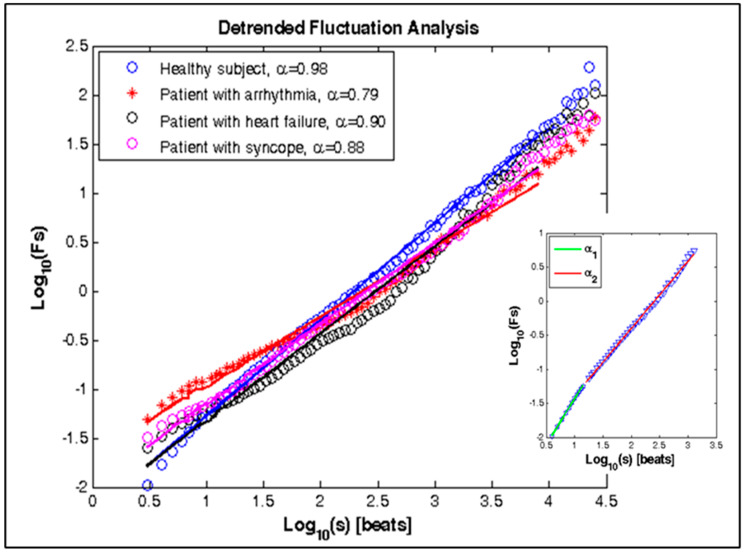
DFA for healthy subject and patients with arrhythmia, heart failure, and syncope.

DFA method. The values of α, α_1_, and α_2_ parameters for the signals studied are shown in Table 3. The values of these parametere decrease in patients with cardiovascular disease (arrhythmia, heart failure, and syncope), as the statistical significance of less than 0.0001. Therefore, this method can distinguish healthy subjects from those with diseases. Figure 8 shows the graphical dependencies of the four studied groups. The graphs are linear, which is evidence of the fractal behavior of the signals.

MFDFA method. The Hurst parameter determines the degree of regularity and the large-scale invariance of the study process. 

**Figure 9:**
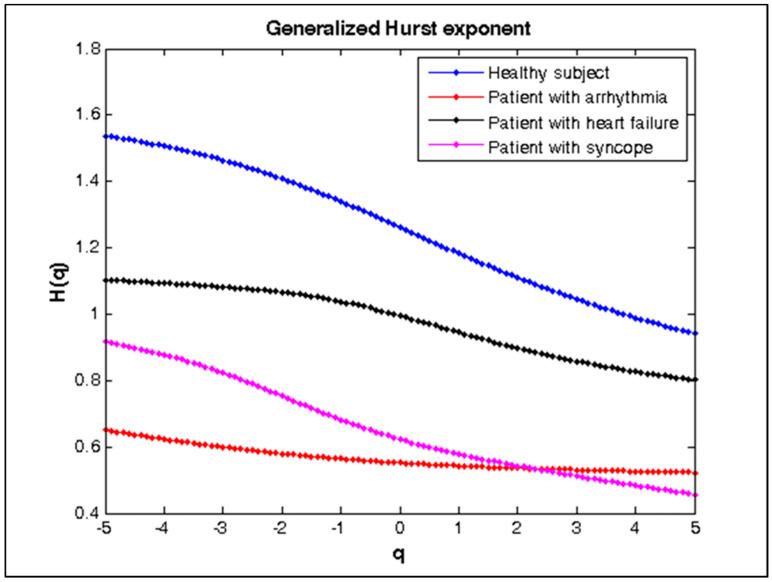
Generalized Hurst exponent for healthy subject and patients with arrhythmia, heart failure, and syncope.

**Figure 10:**
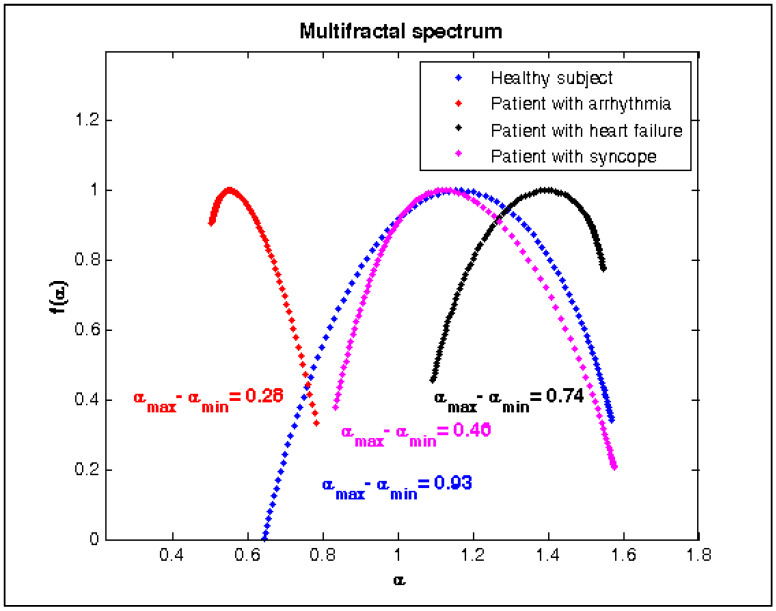
Multifractal analysis for healthy subject and patients with arrhythmia, heart failure, and syncope.

The range of values of the Hurst parameter varies from 1.5 to 0.9 in the case of different values of the q parameter for a healthy subject (Figure 9), therefore these RR intervals have multifractal behavior. In the case of a patient with arrhythmia (Figure 9), the Hurst parameter is almost constant at different values of the q parameter, so this signal has a monofractal behavior. RR interval series of patients with heart failure and syncope (Figure 9) have multifractal behavior concerning the Hurst parameter, but less pronounced compared with the behavior of the healthy subject. Figure 10 shows the multifractal signal spectrum of the studied groups. The healthy subject’s RR signal exhibits a broad spectrum of scaling, indicating that it has multifractal behavior. The signals of patients with arrhythmia, heart failure, and syncope show a narrower range of the multifractal spectrum than that of the healthy subject. The multifractal spectrum of a patient with arrhythmia is almost four times smaller than that of a healthy subject.

## 4. Discussion

### 4.1. Linear Analysis

All Time-Domain analysis parameters examined are within the normal range for healthy subjects. In patients, there is a decrease in RR intervals and in HRV, which is due to a decrease in parasympathetic and increased sympathetic activity.

The SDNN and SDANN parameters reflect the overall effect of autonomic regulation of the heart, therefore their reduction in patients with cardiovascular disease (arrhythmia, heart failure, and syncope) indicates a weakening of the autonomic regulation of the cardiovascular system as a whole (both sympathetic and sympathetic and sympathetic) and reducing the adaptive capacity of the cardiovascular system, which is an adverse factor. The decrease in the values of these two parameters is a very specific sign for the prediction of serious cardiovascular disease, which can endanger the life of the patient [48]. There was a significant decrease in the SDANN parameter (*p* < 0.0001), which was less than 100 ms in all three patient groups (arrhythmia, heart failure, and syncope), indicating a decrease in HRV.

The RMSSD parameter estimates the degree of the difference between two adjacent RR intervals. In the absence of fluctuations in adjacent RR intervals, this parameter tends to zero. The greater the difference between the adjacent intervals (i.e., more pronounced sinus arrhythmia), the higher the RMSSD values are, therefore the more active the relationship of parasympathetic regulation. According to our results, the decrease in the rMSSD quantitative values in the three groups of patients with cardiovascular disease compared with the group of healthy subjects showed a decrease in the parasympathetic nervous system tone and an increase in sympathetic activity. The determined values of this parameter have a statistical significance of *p*-value < 0.0001. Similar information can also be obtained from the pNN50 index, which expresses in % the number of difference values greater than 50 ms. The NN50 parameter also reflects the difference between the adjacent RR intervals, but the main evaluation criteria, in this case, is the difference between two adjacent intervals of more than 50 ms. This situation occurs when there are sudden pauses or faster rhythms. The main reason for the sudden pauses is the dominance of the parasympathetic effects on heart rate. Of the methods based on the analysis of the difference between adjacent NN intervals, the calculation of RMSSD is preferable since it has better statistical properties than NN50 and pNN50.

Geometric methods allow a visual representation of the distribution of RR intervals. It is generally accepted to use a histogram of the distribution of RR intervals, the width of which indicates the values of the RR intervals and the height the frequency of occurrence of these cardio intervals. The shape of the histogram depends on the specific physiological condition of the subject. The healthy people have a histogram similar in appearance to the symmetric Gaussian curve (Figure 3A). The asymmetric shape of the histogram (Figure 3B) shows a violation of the stationary process of regulating the heart rhythm and is observed in transition and pathological conditions in the cardiovascular system, such as arrhythmia. The histogram with a very narrow base and a pointed tip is registered under severe stress and pathological conditions, such as heart failure (Figure 3C). A two-tip histogram (Figure 3D) may be due to the presence of a non-sinus rhythm (atrial fibrillation, extrasystole), as well as to artefacts that appear during electrocardiogram registration. To describe the deviation of the histogram shape from the normal distribution law, geometric estimates are used: HRVti and TINN. The main advantage of geometric methods is that they are poorly influenced by extrasystoles—if for some reason they do not recognize and remove extrasystoles, this will not lead to any significant changes in the result.

Frequency-Domain Analysis. The healthy control group studied has high values of spectral components in all tested frequency bands and a sympathetic balance value corresponding to good health (according to HRV standard).

Higher diagnostic and prognostic value compared to short records is achieved when performing research on 24-hour Holter records.

Studies of patients with arrhythmia show low values of spectral parameters, which can conclude the severity of this widespread heart disease. This is the group with the lowest LF vs. HF ratio (0.81), which shows significantly lower LF values that reflect the sympathetic and parasympathetic activity of the nervous system.

Reduced VLF in combination with multiple reduced spectral components in the LF and HF area indicates patients with heart failure who are at increased risk of future cardiovascular events.

Measured high VLF values in patients (significantly higher than in healthy subjects) with heart disease may be a good predictor of subsequent syncope in the subject; the value of VLF can be used to evaluate patients admitted after a loss of consciousness.

The studies and the results obtained show that realistic diagnostic and prognostic conclusions can be drawn by evaluating the spectral components of HRV.

Time-Frequency Analysis. Time-Frequency spectrograms clearly illustrate HRV values in the three tested frequency bands and can be an effective tool for creating a rapid diagnostic and prognostic picture for the development of a patient’s heart disease. Healthy individuals are characterized by high variability in the three frequency bands represented by a warm-color spectrogram. Arrhythmia recording spectrograms show decreasing trends and then increasing variability. Heart failure records are characterized by average HRV levels in VLF and LF areas and very low HF areas in the spectrogram. Syncope recordings are characterized by high variability in the VLF area and low in the other two regions (represented by blue fields in the spectrogram).

### 4.2. Nonlinear Analysis

The great methodological difficulty in assessing HRV is related to the unstable nature of cardio intervals. Heart rate fluctuations have a self-similarity (fractal characteristics), which is why it is necessary to use mainly long-term ECG records provided by Holter monitoring.

Poincaré’s nonlinear method is a useful tool when rare and sudden abnormalities occur in the background of a monotonous heart rate. This method is suitable for the study of certain cardiovascular diseases, such as arrhythmias, where the methods of statistical and spectral analysis of HRV are uninformative and, in these cases, it is appropriate to use the Poincaré plot estimate. Through this method, the activity of the sympathetic autonomic nervous system in relation to the heart can be evaluated. The scatherogram of a healthy subject has a well-defined ellipse, which means that a certain amount of non-respiratory arrhythmia is added to the respiratory arrhythmia. If the ellipse is in the form of a circle, it means the absence of non-respiratory components of arrhythmia. The lower values of the SD1 and SD2 parameters in patients with arrhythmia, heart failure, and syncope lead to a decrease in HRV, which is a prognostic marker for the presence of cardiovascular disease. This visual method enables doctors to view the entire 24-hour ECG record at a glance and quickly detect cardiovascular disorders if any.

Another promising method for HRV research is the Recurrence plot, which allows the visualization of certain regularities in the complex non-stationary fluctuations of the RR interval series. The method is based on the design and analysis of recurrence diagrams. The point model of the Recurrence plot (Figure 7) in patients with diseases is significantly different from that of healthy subjects. First of all, there is an increase in the number of points, which also increases the number of conditions, the distances between which are smaller than the threshold value, i.e., the value of REC% increases. At the same time, an increase in the length of the diagonal lines is observed, with significantly increased values of Lmax, Lmin, and ENTR. Increasing the length of the diagonal lines leads to a decrease in the chaotic component of the HRV, since the maximum and average length are characterized by an inverse correlation concerning the indicator of the chaotic properties of the studied process—the largest Lyapunov index. An increase in the DET% ratio also indicates a decrease in the signal complexity level. The DET% value is maximum (100%) for a periodic process and minimum (0%) for a stochastic process. The results of the study indicate that Recurrence plots can be good tools for analysing and evaluating HRV time series.

The results obtained by applying the R/S method show that the Hurst exponents tend to be 1.0, therefore the studied groups have fractal behaviour with a high degree of self-similarity. These values of the Hurst parameter indicate that the investigated signals have less variability and less roughness. The results obtained show that this method cannot distinguish healthy subjects from those with the cardiovascular disease since they have no statistical significance (*p* > 0.05). The probable reasons for this result can be the following: the investigated signals are long (24 hours), containing a large number of intervals (about 100,000); the investigated signals are characterized by nonlinear behaviour, and the method is designed for the study of stationary signals. In such cases, the R/S method is only appropriate to check that the signals under study have fractal behaviour, but where a more accurate determination of the Hurst parameter is required, a more accurate statistical method should be applied.

The results of the nonlinear analysis obtained by applying the DFA method show a higher value of the Alpha parameter in healthy subjects compared to patients with cardiovascular disease. Therefore, the level of complexity and the chaotic component decrease and the degree of self-similarity at RR intervals increases in healthy subjects.

The multifractal spectrum of the patients with arrhythmia, heart failure, and syncope is narrower than healthy controls, indicating a significant decrease in nonlinear heart rate measured by HRV. A significant reduction in the width of the multifractal spectrum is a marker of cardiac dysfunction. Patients with cardiovascular disease in terms of spectrum width are examples of monofractal behaviour. The multifractal spectrum of the studied groups is statistically significant (*p* < 0.0001); therefore, healthy subjects may be distinguished from patients with arrhythmia, heart failure, and syncope in terms of multifractal width parameter.

AppEn and SampEn are higher for healthy subjects than patients with arrhythmia, heart failure, and syncope. Higher values of these parameters are evidence that RR interval series for healthy people have stochastic behaviour.

## 5. Limitations

This study has a few limitations. The established HeartAnalyzer was tested with a limited number of Holter data for the individual patient groups and the control group of healthy individuals (total number of analysed 212) provided to us by the Medical University, Varna, Bulgaria. At the moment, the authors are working on the creation of a real cardiology database consisting of Holter recordings obtained from monitoring patients with various cardiac diseases. In their future studies, the authors will provide data from studies conducted on a larger number of Holter records (including from various medical institutions), which will guarantee high reliability of the developed computer system.

## 6. Conclusions

Overall assessment of HRV is aimed at diagnosing the patient’s functional status, the analysis is a non-specific diagnostic method. The evaluation of the totality of its indicators and their dynamics during multiple examinations of the patient allows the diagnostic search to be directed in the right direction and helps to clarify the functional and prognostic components of the clinical diagnosis.

In the current stage of the practical application of the methods of HRV analysis in applied physiology and clinical medicine, the above approaches to the physiological and clinical interpretation of the data allow for solving many problems from the diagnostic and prognostic profile more effectively, to evaluate the functional states, to evaluate monitors for the effectiveness of therapeutic and prophylactic effects, etc. However, the possibilities of this methodology are far from being exhausted and its development continues.

The computer system presented in this article can be used to provide a mathematical interpretation of cardiac patient studies. Through the obtained analyses and results, the HeartAnalyzer can improve the accuracy of diagnosis, assist the treating cardiologist in the right choice of appropriate drugs and thus shorten the time for decision-making by the physician, speed up the healing process and reduce the costs of treating patients. The HeartAnalyzer created is an opportunity to overcome the presence of the subjective factor in making the diagnosis and to bring greater accuracy and objectivity in the conduct of treatment.

The presented HeartAnalyzer can be used for prevention as the parameters of the HRV begin to change before the risk event itself occurs. In this way, the HeartAnalyzer can assist with the early detection of diseases and their prompt treatment. The system can also serve as a predictor of a life-threatening event and enable the patient and physician to overcome the oncoming health crisis.

One of the main advantages of this work is the research on real Holter data, which makes it possible to test the performance of the HeartAnalyzer through cardiac data obtained from patients examined at a medical establishment in Bulgaria.

As a result, it can be expected that having a non-commercial computerized system in place can help improve people’s health and be a prerequisite for improving health.

## Figures and Tables

**Table 1:** Time-Domain HRV analysis results for studied groups.

Parameter	Group 1 (mean ± SD) *n* = 48	Group 2 (mean ± SD) *n* = 56	Group 3 (mean ± SD) *n* = 59	Group 4 (mean ± SD) *n* = 49	Statistical *p*-Value		
					Gr_1,2_	Gr_1,3_	Gr_1,4_
**Statistical measurement**							
MeanRR [ms]	849 ± 28	679 ± 18	880 ± 20	855 ± 35	0.0001	0.0005	0.0001
SDNN [ms]	121.8 ± 21	62 ± 15	72 ± 18	60 ± 15	0.0001	0.0001	0.0001
SDANN [ms]	140 ± 15	64 ± 10	70 ± 12	28 ± 4	0.0001	0.0001	0.0001
pNN50 [%]	14.8 ± 3	13 ± 5	9.1 ± 2	6.7 ± 1	0.03	0.0001	0.0001
RMSSD [ms]	25.8 ± 9	17 ± 2	16 ± 3	12 ± 5	0.0001	0.0001	0.0001
**Geometrical measurement**							
HRVti [numb]	21.8 ± 10	6.2 ± 2.7	1.5 ± 1.2	3.1 ± 1.6	0.0001	0.0001	0.0001
TINN [ms]	493 ± 80	542 ± 70	381 ± 60	52 ± 11	0.002	0.0001	0.0001

**Table 2:** Frequency-Domain HRV analysis results for studied groups.

Parameter	Group 1 (mean ± SD) *n* = 48	Group 2 (mean ± SD) *n* = 56	Group 3 (mean ± SD) *n* = 59	Group 4 (mean ± SD) *n* = 49	Statistical *p*-Value		
					Gr_1,2_	Gr_1,3_	Gr_1,4_
VLF Power [ms^2^]	13226.42 ± 674.12	12602.93 ±984.17	11939.57 ± 489.73	17846.84 ± 692.41	0.0004	0.0001	0.0001
LF Power [ms^2^]	1198.88 ± 562.93	549.98 ± 181.42	411.82 ± 247.79	486.26 ± 164.33	0.0001	0.0007	0.0001
HF Power [ms^2^]	791.03 ±243.18	675.71 ± 269.14	301.93 ± 354.81	534.35 ± 388.96	0.0234	0.002	0.0002
LF Power nu	0.602 ± 0.23	0.449 ± 0.11	0.577 ± 0.19	0.476 ± 0.21	0.0001	*NS* * (0.5393)	0.0059
HF Power nu	0.398 ± 0.19	0.551 ± 0.13	0.423 ± 0.08	0.524 ± 0.195	0.0001	*NS* * (0.3615)	0.0018
LF/HF	1.52 ± 0.57	0.81 ± 0.22	1.36 ± 0.07	0.91 ± 0.68	0.0001	0.0475	0.0001

* NS—Not Significant.

**Table 3:** Nonlinear HRV analysis results for studied groups.

Parameter	Group 1 (mean ± SD) *n* = 48	Group 2 (mean ± SD) *n* = 56	Group 3 (mean ± SD) *n* = 59	Group 4 (mean ± SD) *n* = 49	Statistical *p*-Value		
					Gr_1,2_	Gr_1,3_	Gr_1,4_
**Poincaré plot**							
SD1 [ms]	61.2 ± 10.3	55.5 ± 12.8	49.8 ± 9.9	45.1 ± 11.0	0.014	0.0001	0.0001
SD2 [ms]	218.1 ± 26.2	73.3 ± 10.5	106.2 ± 11.9	96.1 ± 9.2	0.0001	0.0001	0.0001
SD1/SD2	0.31 ± 0.7	0.87 ± 0.11	0.54 ± 0.2	0.52 ± 0.12	0.0001	0.0001	0.0001
**Recurrence plot**							
DET [%]	90.8 ± 0.11	97.9 ± 0.13	99.06 ± 0.09	98.8 ± 0.1	0.0001	0.0001	0.0001
REC [%]	36.3 ± 0.2	43.4 ± 0.5	41.1 ± 0.3	39.5 ± 0.3	0.0001	0.0001	0.0001
L_max_ [beats]	58 ± 12	136 ± 22	305 ± 31	104 ± 11	0.0001	0.0001	0.0001
ENTR	3.20 ± 0.3	3.48 ± 0.4	4.12 ± 0.1	3.80 ± 0.3	0.0001	0.0001	0.0001
**R/S method**							
Hurst	0.98 ± 0.07	0.95 ± 0.04	0.96 ± 0.05	0.94 ± 0.13	0.06	0.09	0.06
**Detrended Fluctuation Analysis**							
α	0.98 ± 0.03	0.77 ± 0.05	0.86 ± 0.06	0.81 ± 0.07	0.0001	0.0001	0.0001
α_1_	1.16 ± 0.04	0.79 ± 0.04	0.89 ± 0.05	0.82 ± 0.06	0.0001	0.0001	0.0001
α_2_	0.91 ± 0.03	0.68 ± 0.03	0.75 ± 0.04	0.72 ± 0.04	0.0001	0.0001	0.0001
**Multi-Fractal Detrended Fluctuation Analysis**							
Δα=α_max_−α_min_	0.956 ± 0.05	0.281 ± 0.01	0.773 ± 0.03	0.494 ± 0.04	0.0001	0.0001	0.0001
**Entropies**							
AppEn	1.57 ± 0.19	1.29 ± 0.25	1.08	1.32	0.0001	0.0001	0.0001
SampEn	1.53 ± 0.22	1.14 ± 0.21	1.17	1.25	0.0001	0.0001	0.0001
